# The unmet need of electrolyte supplements

**DOI:** 10.1093/ndt/gfaf161

**Published:** 2025-08-21

**Authors:** Susana Carvajal Arjona, Antonio Cabrera Cantero, David van Bennekom, Etienne Cosyns, Renée de Wildt, Virginia Guillon

**Affiliations:** Hipofam, Association for Information and Research on Familial Hypomagnesemia, Barcelona, Spain; FEDERG, Federation of European Associations of Patients Affected by Rare and/or Genetic Kidney Diseases, Brussels, Belgium; The European Patient Advocacy Group (ePAG), Working Group Tubulopathies, ERKNet, Heidelberg, Germany; Hipofam, Association for Information and Research on Familial Hypomagnesemia, Barcelona, Spain; The European Patient Advocacy Group (ePAG), Working Group Tubulopathies, ERKNet, Heidelberg, Germany; AIRG-E, Asociación para la información y la investigación de las enfermedades renales genéticas en España, Barcelona, Spain; The European Patient Advocacy Group (ePAG), Working Group Metabolic Nephropathies, ERKNet, Heidelberg, Germany; Nierpatiënten Vereniging Nederland, Bussum, The Netherlands; The European Patient Advocacy Group (ePAG), Working Group Tubulopathies, ERKNet, Heidelberg, Germany; AIRG-Belgique, Association pour l'Information et la Recherche sur les maladies Rénales Génétiques, Brussels, Belgium; The European Patient Advocacy Group (ePAG), Working Group Tubulopathies, ERKNet, Heidelberg, Germany; Nierpatiënten Vereniging Nederland, Bussum, The Netherlands; The European Patient Advocacy Group (ePAG), Working Group Tubulopathies, ERKNet, Heidelberg, Germany; Association Gitelbart, Paris, France

Electrolytes are essential for the management of various kidney pathologies, both hereditary and acquired, including rare diseases [1, 2]. In this specific context, they do not represent simple supplementations, but true treatments. The doses that need to be used often exceed doses used for standard supplementation. Limited availability and the frequent need to use compounded hospital formulations complicate access to these treatments. Many electrolyte formulations are often not well suited for treatment, are unpalatable, and sometimes poorly tolerated, particularly in children. Inconsistent regulations across European countries result in variable labelling of electrolyte solutions as food supplements or actual medicines, which produces unequal healthcare access and often a significant financial burden for patients with rare kidney diseases and their families. Overall, this may lead to an increased risk of defective treatment or even interruption, even though electrolyte supplement solution are commonly required for long periods of time, or even lifelong.

The European Patient Advocacy Group (ePAG) of the Tubulopathies Working Group conducted an analysis derived from a survey across various ERKNet expert centres (ERKNetEC). The primary objective was to provide an overview of the access to electrolyte treatments in different member states, to identify instances of inequity and any lack of national insurance coverage, and to assess the availability of suitable formulations for children. The ultimate goal of this work is to improve and standardize care for patients with rare kidney diseases at the European level.

**Figure 1:**
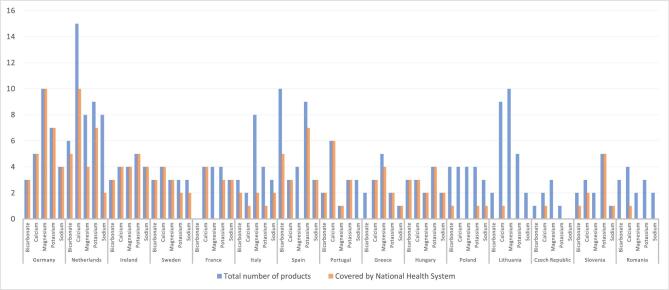
Total number of electrolyte products available per country and the corresponding number covered by the respective National Health System.

The data presented in this report were collected from professionals (specialists in hereditary kidney disorders and electrolyte management) affiliated in 15 different ERKNetEC, encompassing nine Western European countries (France, Germany, Spain, Portugal, Italy, the Netherlands, Ireland, Greece, and Sweden) and six Eastern European countries (Romania, Hungary, Slovenia, Lithuania, Poland, and the Czech Republic) Fig. 1. The final report was reviewed and is supported by the boards of the Working Groups Genes & Kidney of the ERA and Inherited Kidney Diseases of the ESPN.

This report focuses on five different electrolytes: bicarbonate, calcium, magnesium, potassium, and sodium.

Sodium bicarbonate is usually used as a compound to reduce gastric acidity but represents a key therapy for treating metabolic acidosis in chronic kidney diseases and tubular disorders. It is an inexpensive, widely accessible product available in all surveyed expert centres. In most countries, sodium bicarbonate is reimbursed by the National Health Systems, but exceptions include Greece, Lithuania, the Czech Republic, and Romania. Specific formulations for children are lacking in several countries: while Spain offers five child-adapted products, six countries (Germany, Greece, Italy, Sweden, Lithuania and Poland) have only one, and six countries (Netherlands, Ireland, Hungary, Portugal, Czech Republic and Slovenia) lack paediatric-specific formulations entirely. Since sodium bicarbonate is commonly prescribed for children and is inexpensive, it is unreasonable not to provide formulations adapted for children.

Calcium supplements were generally available in all countries included in the surveyed ERKNetEC. In 9 of the 15 countries, at least three calcium products are covered by the respective National Health Systems. However, Poland, despite offering four products, provides no financial coverage. Four countries have only one reimbursed product, while the Netherlands stands out with 15 products, of which 10 are reimbursed. Paediatric calcium formulations are largely unavailable, with only six countries (Netherlands, Greece, Ireland, Sweden, Portugal, and Romania) offering at least one product for children. For adults, tablets are the most common form, with syrups and powders less frequently available.

Magnesium is essential for managing hereditary and acquired hypomagnesaemia. Magnesium deficiency can lead among others, to neurological manifestations, fatigue, and muscle cramps, requiring long-term or even lifelong supplementation for many patients [3]. The analysis of magnesium availability across the 15 ERKNetEC reveals significant disparities. The number of products ranges from 10 in countries such as Germany and Lithuania to only one in Portugal. Only 53% of countries offer magnesium products that are covered by National Health Systems; Germany covers all 10 available products, although in many cases, coverage is granted only after individual negotiations with insurance companies. In the absence of such negotiations, insurers often classify these products as ‘lifestyle medications’, excluding them from coverage. Countries such as Spain, France, and Poland provide no coverage at all. Unfortunately, magnesium products are commonly marketed as supplements for athletes, which is often misleading and decreases the likelihood of being reimbursed for patients with rare diseases. Although most countries have at least one option for children, two-thirds of centres report only two or fewer options suitable for the treatment of children, and Slovenia reports none. The types of formulation are also limited, with tablets and capsules dominating (48%), whereas syrups and powders that are suitable for therapy in children represent only 18% and 16% of products, respectively. Effective supplementation also depends on the bioavailability of magnesium, which is highly variable across formulations [4].

Potassium metabolism disorders are among the most common electrolyte disturbances in clinical practice, ranging from mild diuretic-induced hypokalaemia to severe, life-threatening hyperkalaemia. Both hypo- and hyperkalaemia disrupt cell membrane polarization, with significant effects on neuromuscular excitability and potentially severe cardiovascular complications. Conditions such as Gitelman, Bartter, and Liddle syndromes often result in hypokalaemia, which, in severe cases, can cause arrhythmias, muscle weakness and paralysis [1, 2]. Potassium supplementation is essential for managing these disorders and reducing symptoms and cardiovascular risks. Analysis of potassium product availability across the different centres revealed significant variability, with some countries offering up to nine products (e.g. the Netherlands and Spain) and others as few as one or two (e.g. the Czech Republic and Greece). While 80% of countries provide reimbursement for potassium products, disparities remain: countries such as Lithuania and Romania offer no funded options, while Germany, Spain, and the Netherlands finance up to seven products. Formulations suitable for children are insufficient: 40% of countries lack suitable options for children (e.g. Germany, Hungary, and Portugal), and of the remaining 60%, most offer only one or two choices. In terms of different formulations, tablets dominate (28%), followed by injectables (27%), syrups (16%), and powders (10%), with the last being most suitable for young children.

Sodium supplement availability across the 15 ERKNetEC shows generally good access, with the number of products ranging from eight in the Netherlands to only one in Slovenia and Greece. While most countries provide sodium products covered by their National Health Systems, exceptions include Portugal, Lithuania, and Romania, which offer no funded options. Paediatric-friendly presentations are notably scarce. Of the 14 countries with data, only five have products suitable for children (the Netherlands, Italy, Sweden, Poland, and Spain), and in three of these, only one option is available. Vials containing concentrated sodium solution for IV injection that are used in the hospital settings, can be used to fractionate doses in children, but are not always available and often unpalatable. In terms of types of formulations, nearly 50% of products are injectables, with syrups and tablets comprising 21% and 19%, respectively.

In conclusion, while electrolyte products for adults are generally available in various formulations, there are significant gaps in paediatric-specific formulations and in the financial coverage by different National Health Systems. Our data reveal substantial variability across countries, with Eastern European countries offering less choices and financial coverage. This disparity is most pronounced for magnesium, bicarbonate, and sodium.

All electrolytes need to increase substantially in their availability to achieve equitable care. Due to ongoing urinary losses in tubular kidney diseases, electrolyte supplements typically need to be taken multiple times a day, complicating the daily life of patients and their families. There is a clear unmet need for the provision of delayed-release formulations that not only simplify the life of the patients but also help maintain better sustained control of the electrolyte abnormalities.

Lack of public funding in lower-income countries exacerbates the financial burden on families, highlighting the need for socioeconomic considerations when evaluating access to treatments. It is not uncommon for patients to spend 100–150 euros per month, which, in some countries, corresponds to 10%–15% of the average monthly net salary. Options of formulations adapted for paediatric care are especially limited for calcium and sodium, while for magnesium, greater product diversity is needed to address patient tolerance and specific needs.

Most importantly, these products need to be officially classified in many countries as essential therapies for rare kidney diseases and not as nutritional supplements. There are ∼25 different renal tubular disorders. Assuming an average prevalence of 1 in 100 000, the estimated cumulative prevalence is ∼1 in 4000. Based on this approximation, these disorders collectively affect around 100 000 individuals across Europe.

Data from ERKReg [5], which currently includes ∼2500 paediatric and adult patients with tubulopathies, highlight the clinical need for equitable access to essential therapies across Europe. Our survey highlights the need for a coordinated action across Europe.

We urge European and national health authorities to address these disparities and to promote equitable care for all patients. More specifically, we recommend:

- increasing the availability of electrolyte supplements in under-resourced countries
- expanding adapted formulations for children
- diversifying compositions to improve patient tolerance
- ensuring equitable healthcare coverage for electrolytes across all European countries
